# Mining sequence variations in representative polyploid sugarcane germplasm accessions

**DOI:** 10.1186/s12864-017-3980-3

**Published:** 2017-08-09

**Authors:** Xiping Yang, Jian Song, Qian You, Dev R. Paudel, Jisen Zhang, Jianping Wang

**Affiliations:** 1 Department of Agronomy, University of Florida, Gainesville, FL 32610 USA; 2 FAFU and UIUC-SIB Joint Center for Genomics and Biotechnology, Haixia Institute of Science and Techonology, Fujian Agriculture and Forestry University, Fuzhou, Fujian 350002 China; 3 Genetics Institute, Plant Molecular and Biology Program, University of Florida, Gainesville, FL 32610 USA

**Keywords:** Sugarcane, *Saccharum* Complex, Sequence variations, Genotyping by sequencing (GBS), Polyploid, Sequence features, Sequence divergence

## Abstract

**Background:**

Sugarcane (*Saccharum* spp.) is one of the most important economic crops because of its high sugar production and biofuel potential. Due to the high polyploid level and complex genome of sugarcane, it has been a huge challenge to investigate genomic sequence variations, which are critical for identifying alleles contributing to important agronomic traits. In order to mine the genetic variations in sugarcane, genotyping by sequencing (GBS), was used to genotype 14 representative *Saccharum* complex accessions. GBS is a method to generate a large number of markers, enabled by next generation sequencing (NGS) and the genome complexity reduction using restriction enzymes.

**Results:**

To use GBS for high throughput genotyping highly polyploid sugarcane, the GBS analysis pipelines in 14 *Saccharum* complex accessions were established by evaluating different alignment methods, sequence variants callers, and sequence depth for single nucleotide polymorphism (SNP) filtering. By using the established pipeline, a total of 76,251 non-redundant SNPs, 5642 InDels, 6380 presence/absence variants (PAVs), and 826 copy number variations (CNVs) were detected among the 14 accessions. In addition, non-reference based universal network enabled analysis kit and Stacks de novo called 34,353 and 109,043 SNPs, respectively. In the 14 accessions, the percentages of single dose SNPs ranged from 38.3% to 62.3% with an average of 49.6%, much more than the portions of multiple dosage SNPs. Concordantly called SNPs were used to evaluate the phylogenetic relationship among the 14 accessions. The results showed that the divergence time between the *Erianthus* genus and the *Saccharum* genus was more than 10 million years ago (MYA). The *Saccharum* species separated from their common ancestors ranging from 0.19 to 1.65 MYA.

**Conclusions:**

The GBS pipelines including the reference sequences, alignment methods, sequence variant callers, and sequence depth were recommended and discussed for the *Saccharum* complex and other related species. A large number of sequence variations were discovered in the *Saccharum* complex, including SNPs, InDels, PAVs, and CNVs. Genome-wide SNPs were further used to illustrate sequence features of polyploid species and demonstrated the divergence of different species in the *Saccharum* complex. The results of this study showed that GBS was an effective NGS-based method to discover genomic sequence variations in highly polyploid and heterozygous species.

## Background

Next generation sequencing (NGS) can generate millions of sequencing reads in parallel in a single run, greatly changing the landscape of genetics by determining the genotypes of interest at an unimaginable speed [1]. The efficiency of NGS has been further improved through multiplex sequencing by tagging different DNA samples using unique barcodes (short DNA sequences) [2]. NGS has been widely used for whole genome sequencing and re-sequencing to discover sequence variations, genetic linkage analysis, quantitative trait loci (QTL) analysis, and genome wide association study (GWAS) [3–5]. This approach is suitable for species with a small genome, but has been a challenge for sugarcane with an estimated genome size up to 10 Gbp and a total of 100–130 chromosomes [6].

To capture genome-wide sequence variations and developing genetic markers for species with complicated genomes, it would be feasible to sequence a subset of the genome after reduction of genome complexity. Several methods that utilize NGS to detect sequence variations by sequencing a subset of genomes have previously been discussed [7]. Among these methods, target enrichment and restriction-enzyme-digestion methods are efficient, powerful, and widely applied to produce a representative subset of genomes for genome-wide variation mining.

Target enrichment methods include polymerase chain reaction (PCR)-based target amplification, capture-by-circularization, and hybrid capture methods [8, 9]. In these methods, primers or probes, designed according to prior available reference sequences, were used to fish out the target regions of interests or representative genomic regions to narrow down or reduce the complexity of the whole genome. Restriction-enzyme-digestion methods include reduced-representation libraries (RRLs), complexity reduction of polymorphic sequences (CRoPS), restriction-site-associated DNA sequencing (RAD-seq), multiplexed shotgun genotyping (MSG) and genotyping by sequencing (GBS) [7].

GBS is a technology enabled by NGS and could reduce genome complexity using restriction enzymes. Compared to the target capture methods, GBS is easy, quick, highly reproducible, and is able to detect genome-wide sequence variations without depending on prior sequence information [2]. GBS has been widely used for marker detection, linkage mapping, QTL analysis, GWAS, genomic selection and molecular breeding in more than 300 species [2, 10–16].

Although these NGS-based methods are popular and powerful, it is daunting to perform them for genetic variation detection in sugarcane due to its large genome size and high heterozygosity, which requires high sequence coverage to resolving sequence variations with different dosages. With continuing efforts from the sugarcane research community, several projects were reported by exploiting the advantages of NGS after reducing genome complexity of sugarcane. For example, in 2013, Bundock et al. had used probe hybridization capture sequencing (HCS) to enrich sugarcane target regions in comparison with the whole-genome shortgun sequencing approach [17]. The results showed that the enrichment of target regions through HCS was 10–11-fold higher in sequence depth than the whole genome sequencing approach. HCS was approved to be greatly helpful for sequence variation detection by using NGS reads from limit sequencing resource. Grativol et al. (2014) applied methyl filtration with McrBC endonuclease digestion to enrich gene-enrichment regions of the sugarcane genome [18]. Compared to the unfiltered method, the coverage on sorghum coding sequences (CDS) was at least 36% higher with the methylation filtration method, which covered 134 X of gene regions of the monoploid sugarcane genome. Recently, HCS was used in our laboratory to assay 12 representative sugarcane germplasm accessions, in which 55,946 probes were designed to target a total of 6 Mb of the sugarcane genome, and approximately 1.2 million single nucleotide polymorphisms (SNPs) were detected subsequently [19]. Very recently, Balsalobre et al. (2017) applied GBS for sugarcane linkage map construction and QTL analysis [20], which further advanced application of NGS enabled technologies for sugarcane sequence variation calling.

*Saccharum* complex is a set of interbreeding species including sugarcane’s close relatives (Mukherjee 1957), considered as the primary gene pool leading to modern sugarcane cultivar development. It’s critically important to mine sequence variations, assesse sequence divergence, and identify the sequence variants controlling critical agronomic traits in *Saccharum* complex, which will help research community better utilize the genetic resources. Moreover, with the development of NGS, it is essential to explore and evaluate different high throughput genotyping methods at this NGS enabled genomics era for future choosing suitable method specifically for a large scale application. The objectives of this research were 1) to establish the pipelines for GBS data analysis in representative and diverse *Saccharum* complex accessions 2) to call the sequencing variations in the *Saccharum* complex; and 3) to investigate the sequence features and genetic relationships of the 14 accessions by using genome-wide SNPs. Our results demonstrated that GBS is an effective way to discover genomic sequence variations in highly polyploid and heterozygous species, which will facilitate further genetic and genomic studies for polyploid sugarcane improvement.

## Results

### Sequencing, mapping, and reads coverage estimation

**Fig. 1:**
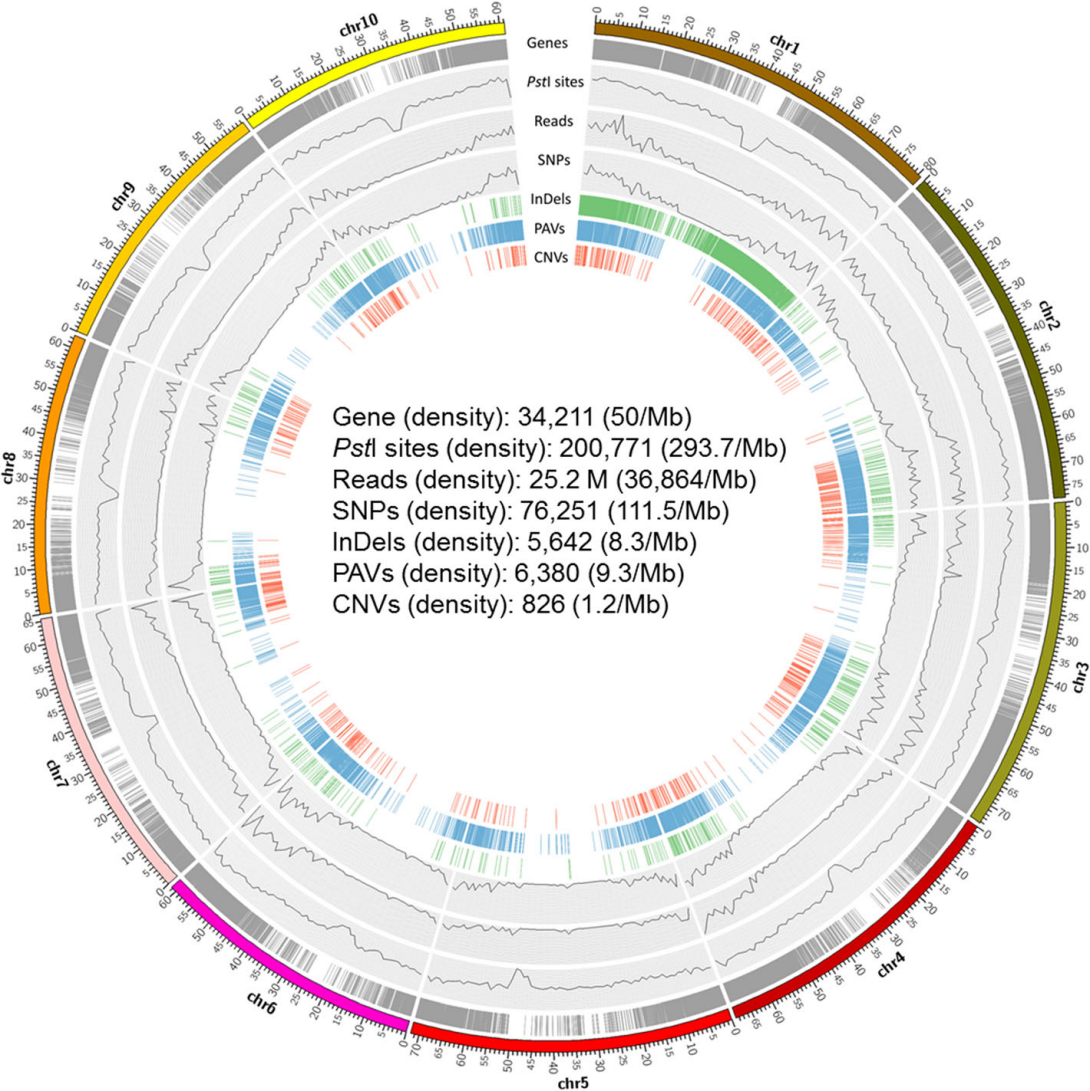
Overview of genome-wide distribution of sorghum gene models, *Pst*I cutting sites, uniquely mapped clean reads from 14 *Saccharum* complex, and different types of sequence variants along the sorghum genome

In total, 103.6 million 100-bp single end reads were generated from GBS of the 14 accessions. After filtering, 78.3 million reads (74%) remained as high quality clean reads with one of the barcodes and the *Pst*I cutting site. To evaluate the efficiency of two widely used aligners, Bowtie 2 and BWA-backtrack, we aligned 13 million clean reads from *Saccharum* hybrid CP88–1762 to sorghum genome 3.0 by using Bowtie 2 and BWA-backtrack, respectively. The percentages of uniquely mapped reads were 33.3% for Bowtie 2 and 13.8% for BWA-backtrack. Bowtie 2 generated much more uniquely mapped reads and was more suitable as the aligner for downstream analyses for sugarcane (see Discussion). Uniquely mapped reads for each accession ranged from 1.2 to 4.3 million, and the percentages of the uniquely mapped reads ranged from 30.0% to 35.8% (Table 1). Among the uniquely mapped reads, most of them (83.3% on average) were mapped to sorghum genic regions, and localized on two distant chromosome arms (Fig. 1). The number of mapped reads decreased as the mapping location got close to the centromere region.

**Table 1:** Species, ploidy levels, clean reads, uniquely mapped reads, uniquely mapped reads in genic regions and estimated depth per allele of the 14 accessions

Clone name	Species	Ploidy level	Reads in million			Estimated number of reads per allele dose
			Clean reads	Uniquely mapped reads (% of clean reads)	Uniquely mapped reads in genic regions (% of uniquely mapped)	
IND81–14	*S. spontaneous*	6x	4.5	1.4 (33.1)	1.2 (85.7)	7.0
US57–060	*E. rufipilu*	6x	3.9	1.3 (33.3)	1.0 (76.9)	5.8
US61–037	Unknown	6x	3.6	1.2 (33.3)	1.0 (83.3)	5.8
Kalimpon	*E. procerum*	6x	4.0	1.2 (30.0)	1.0 (83.3)	5.8
SES196	*S. spontaneum*	8x	4.0	1.3 (32.5)	1.1 (84.6)	4.8
TekchaOk	*S. sinense*	8x	4.1	1.3 (31.7)	1.1 (84.6)	4.8
NG57–054	*S. robustum*	8x	6.2	2.1 (33.9)	1.8 (85.7)	7.9
P-MAG-84	*S. officinarum*	8x	4.2	1.4 (33.3)	1.2 (85.7)	5.3
Pathri	*S. barberi*	8x	6.0	1.9 (31.7)	1.6 (84.2)	7.0
NG96–024	*S. officinarum*	8x	4.2	1.4 (33.3)	1.2 (85.7)	5.3
Q050	*Saccharum* hybrid	12x	4.3	1.4 (35.8)	1.0 (83.3)	2.9
R570	*Saccharum* hybrid	12x	5.0	1.6 (32.0)	1.4 (87.5)	4.1
CP95–1039	*Saccharum* hybrid	12x	11.3	3.7 (32.7)	3.2 (86.4)	9.3
CP88–1762	*Saccharum* hybrid	12x	13.0	4.3 (33.1)	3.7 (86.0)	10.8
Average			5.6	1.8 (32.4)	1.5 (83.3)	6.2

To evaluate the sequence depth of the GBS data, in silico *Pst*I digestion of the sorghum genome 3.0 [21] was conducted. A total of 200,771 cut sites were present in the sorghum genome. Among 200,781 completely digested fragments, 32,340 had a size in a range of 51–500 bp, which presumably should be the majority of targeted fragments enriched during the GBS procedure [2, 22]. The 32,340 in silico *Pst*I digested fragments were evenly distributed in sorghum chromosomes except in the region of centromeres according to the sorghum genome (Fig. 1). Among the 32,340 digested fragments, 14,267 (44.1%) were located in the gene model sequences. Since only the genic regions between sugarcane and sorghum shared high similarity, the uniquely aligned reads in genic regions of each accession according to the sorghum genome was used to estimate the GBS read depth. The estimated depth per allele dose ranged from 2.9 to 10.8 (Table 1).

### Sequence variants calling

**Fig. 2:**
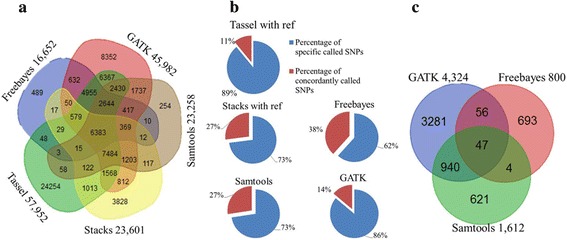
The number of variants called in the 14 sugarcane germplasm accessions. **a** Venn diagram showing overlapping SNPs among five genome reference-based callers, Tassel, Stacks, Samtools, GATK, and Freebayes. **b** Percentage of specific SNPs and concordant SNPs of the total SNPs called by five genome reference-based callers, Tassel, Stacks, Samtools, GATK, and Freebayes. **c** Venn diagram showing overlapping short insertions or deletions (InDels) among three reference-based callers, Samtools, GATK, and Freebayes

With a minimum depth setting of 35 reads at each SNP locus per sample, 57,952, 23,601, 23,258, 45,982, and 16,652 SNPs were called by using reference based callers, Tassel, Stacks, Samtools, GATK, and Freebayes, respectively (Fig. 2a). Among these called SNPs, 82.2, 81.9, 85.2, 84.4 and 91.2% were in genic regions for Tassel, Stacks, Samtools, GATK and Freebayes, respectively. In total, 76,251 non-redundant SNPs were called and 6383 of them were SNPs concordantly called by all the five callers. The percentage of concordantly SNPs ranged from 11% in Tassel to 38% in Freebayes (Fig. 2b), reflecting the different stringency among the five SNP callers. In addition, 34,353 and 109,043 SNPs were called by the non-reference based callers, universal network enabled analysis kit (UNEAK) pipeline in Tassel and Stacks de novo, respectively. In total, 1612, 4324, and 800 InDels were called by using Samtools, GATK, and Freebayes, respectively, with a total of 5642 non-redundant InDels (Fig. 2c). The percentages of InDels in genic regions were 45.2%, 40.4%, and 75.6% for Samtools, GATK, and Freebayes, respectively. For presence-absence variations (PAVs) and copy number variations (CNVs) identification among the 14 accessions, only target regions with a fragment size from 51 to 500 bp after in silico *Pst*I digestion of the sorghum genome were used. In total, 6380 PAVs and 826 CNVs were identified (Fig. 1), in which 83.0% and 90.3% were in genic regions, respectively.

### Sequence depth evaluation for genotype calling and validation

**Fig. 3:**
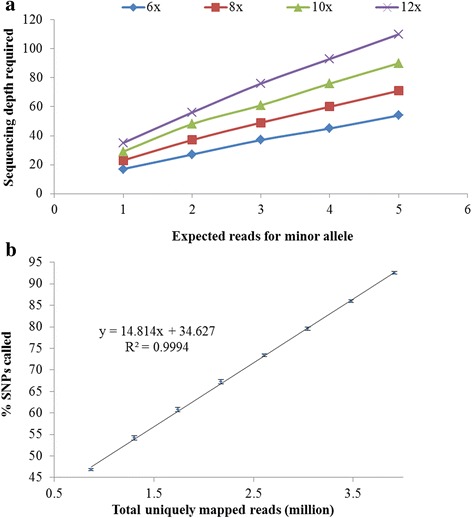
Simulation of read depth for single nucleotide polymorphism (SNP) calling. **a** Relationship between the expected number of reads and the sequencing depth required for minor alleles of single dose SNPs with different ploidy levels, hexaploid (6×), octoploid (8×), decaploid (10×), and dodecaploid (12×). **b** The average percentages of SNPs called with randomly sub-sampled read sets (20, 30, 40, 50, 60, 70, 80, and 90%) compared to the whole read set. The standard deviation of 10 times subsampled results were labeled as bars at the data points

The sequence depth required to obtain the expected number (e.g., 1 to 5) of reads with a probability higher than 95% for minor alleles at SD SNPs locus was estimated at different ploidy levels (Fig. 3a). To obtain the same number of reads in different accessions, a much higher sequence depth was needed for dodecaploid than for hexaploid. For example, the minimum sequence depth estimated would be 27 for hexaploid, 37 for octoploid, 48 for decaploid, and 56 for dodecaploid to obtain two reads for the minor allele. Based on the results, a sequence depth of 56 could achieve two reads for *Saccharum* species with 95% probability, even for *Saccharum* hybrids with up to 12 sets of chromosomes.

The total uniquely mapped reads of a hybrid accession, CP88–1672, was 4.3 million, which had the highest average read depth per allele dose out of the 14 accessions. Eight sub-sample read sets were generated by sampling 20, 30, 40, 50, 60, 70, 80, and 90% of the 4.3 million reads. Uniquely mapped reads of the eight sub-sample read sets ranged from 0.9 million (20%) to 3.9 million (90%). SNPs were called from the eight sub-sample read sets. The percentages of the SNPs out of the overall SNPs (called based on all 4.3 million mapped reads) increased as the sequence depth increased following a linear model (Fig. 3b). Based on the established linear model, 3.7 million (86%) uniquely mapped reads are required to call 90% of expected number of SNPs from the whole read set (4.3 million) and its corresponding sequencing depth was 50.4. Therefore, a sequence depth of 56 (higher than 50.4), was set for filtering SNPs to ensure the presence of two reads for each minor allele at a SD SNP locus and to call more than 90% of the expected SNPs in dodecaploid.

In total, 16 SNPs (Table 2) were randomly chosen for validation from the SNP set after filtering with a minimum sequence depth of 56 per sample. Out of the 16 SNPs, 15 were verified with correct SNP types called in the eight accessions. The percentage of true SNPs is 93.8%. One of the 16 SNPs (SNP4 in Table 2) did not get verified. With close examination of the non-validated SNPs, sequencing quality was poor around the SNP locus because it was at one end of the PCR amplicon. New primers are needed for further validation of this SNP.

**Table 2:** Positions in the sorghum genome and primer sequence of the 16 single nucleotide polymorphisms (SNPs) chosen for PCR amplification and subsequent Sanger sequencing validation (Chr = Chromosome)

Code	SNP type	Caller^a^	Chr.	Forward sequence	Reverse sequence
SNP1	T/A	F, G, Sa, St, T	2	ACGTCAAAAGCTTCGCAGGA	GGCAAAGAAAGCACTGAAGG
SNP2	C/T	F, G, Sa, St, T	2	GCCTGAGATGGCACAAAAAT	CACTGTTTTGCATCCTTTGC
SNP3	G/A	F, G, Sa, St, T	3	CACCACCCACCAACCTAAGC	TTCTTTGCAGTGACCCAGTG
SNP4	A/T	T	3	GGAAGGGCAAGGACTCCTAC	GAAGCCCAGGGACATTGTTA
SNP5	T/C	F, G, Sa, St, T	3	GCTCGCTACAGGGCATAAAG	ACCGTGGTGCTTTTAGAGCT
SNP6	T/C	F, G, Sa, St, T	3	GGGAACCAAAAAGGAGGGTA	GCATACCCTCAAAGGAGTTG
SNP7	C/T	F, G, Sa, St, T	4	GCTCCCAAAGACCATGACAG	TGTCGTGCAGAAGCCAATTA
SNP8	G/A	F, G, Sa, St, T	4	GCTCCCAAAGACCATGACAG	TGTCGTGCAGAAGCCAATTA
SNP9	G/A	F, G, T	4	CATCCTGGTTTTCAGGGGTA	TTGCGGAGGATATGCAGAGT
SNP10	A/G	F, G, Sa, St, T	5	ATGAACGCTTTCCAGATGCT	TCTCACCTGGTGCTCCTTCT
SNP11	C/T	G, Sa, St, T	5	TCAGCCTGGTGAGTTTCCTT	TGTAAACTGGGGAGGACCAG
SNP12	C/T	T	5	TGCAGGTGCTTGCTGAGTAT	AAGCAAGATTGTGCCCTTCA
SNP13	C/A	F, G, Sa, St, T	5	TCTGGATGGGGTACTGGGTA	CCCGGCTAAAGACAATACGA
SNP14	A/G	F	6	CCTGTCCTTAGTGTCTGCAT	CAAGCAAGCAAGTTCCCATT
SNP15	C/T	F, G, Sa, St, T	6	CCTGTCCTTAGTGTCTGCAT	CAAGCAAGCAAGTTCCCATT
SNP16	G/A	F, G, T	6	AAAACATTGAAAGCAGATGC	GCATGTGGCATGTAATGAGG

^a^SNPs were called by reference-based callers: *F* Freebayes, *G* GATK, *Sa* Samtools, *St* Stacks, *T* Tassel

### Heterozygosity, single dose SNPs, and transition/transversion ratios

**Fig. 4:**
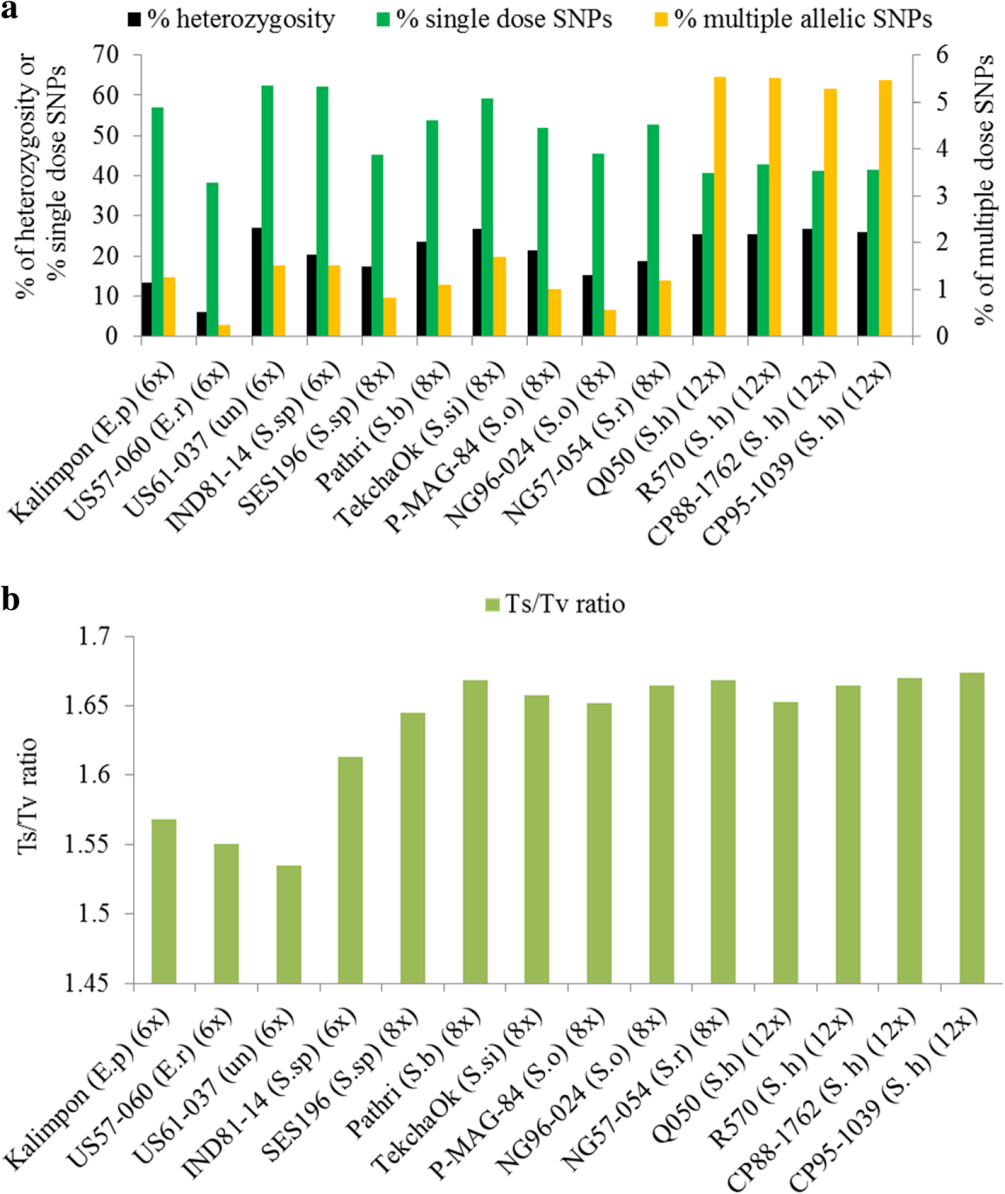
Heterozygosity, genotype, and transition/transversion (Ts/Tv) ratio at single nucleotide polymorphism (SNP) loci in sugarcane. **a** The percentages of heterozygosity, the percentages of multiple allelic SNPs, the percentages of single dose SNPs; **b** Ts/Tv ratios

Based on 13,529 concordantly called SNPs by GATK and Freebayes, the percentages of heterozygosity, SD SNPs, and multiple allelic SNPs, in addition to transition/transversion (Ts/Tv) ratios were characterized for each of the 14 accessions (Fig. 4). The percentages of heterozygosity ranged from 6.1% (for an *Erianthus* clone) to 27.1% (for an unknown clone, most likely a hybrid between *Erianthus* and *Saccharum*) among the 14 accessions. The percentages of SD SNPs ranged from 38.3 to 62.3%. The averages of the SD SNP percentage were 54.9, 51.4, and 41.6% for hexaploids, octoploids, and dodecaploids, respectively, showing a decreasing trend as the ploidy levels increasing. Small portions of concordantly called SNPs were multiple allelic SNPs, ranging from 0.26 to 5.5% among the 14 accessions. Except for four sugarcane hybrids, the percentages of SD SNPs and multiple allelic SNPs were correlated with the percentage of heterozygosity with a correlation coefficient of 0.76, and 0.80, respectively. For the four sugarcane hybrids, the percentages of heterozygosity, the percentages of SD SNPs and the percentages of multiple allelic SNPs were quite similar to each other (Fig. 4a), with an average of 25.8, 41.6, and 5.4% respectively. Among the 14 accessions, Ts/Tv ratios were significantly different between 11 *Saccharum* accessions and the two *Erianthus* accessions (t-test *P* < 0.05), with an average ratio of 1.66 and 1.55 respectively, reflecting sequence variance specificity of the two genera (Fig. 4b). The average Ts/Tv ratio was 1.63 for the 14 accessions.

### Sequence divergence

**Fig. 5:**
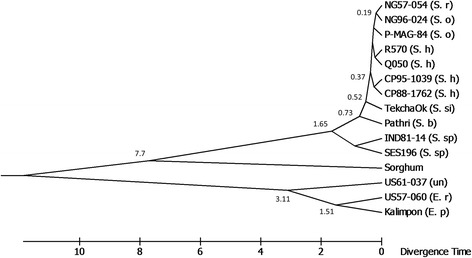
A divergent time tree of the 14 accessions and sorghum generated by maximum likelihood method. The unit for the number at the branch of the tree indicates divergence in million years. Abbreviation in bracket: *S. r* = *S. robustum*; *S.* o = *S. officinarum*; *S.* h = *Saccharum* hybrid; *S. b* = *S. barberi*; S. si = *S. sinense*; *S. sp.* = *S. spontaneum*; *E. r* = *E. rufipilu*; *E. p* = *Erianthus procerum*, and un = unknown

Genetic relationships and divergence time were investigated based on 4879 genome-wide SNPs, concordantly called by all the five reference-based methods with a minimum sequence depth of 56 per locus per sample. The *Erianthus* accessions were clustered as a distinct group, highly divergent from other *Saccharum* accessions (Fig. 5). The unknown accession with undetermined species name (US61–037) was grouped with the *Erianthus* accessions, suggesting their close relatedness. In the *Saccharum* accessions, the divergence of them from their common ancestor followed a decreasing order: *S. spontaneum* > *S. barberi* > *S. sinense* > *Saccharum* hybrids > *S. officinarum*/*S. robustum*. Among the 14 accessions, *Erianthus* diverged from other *Saccharum* species in more than 10 million years ago (MYA) (Fig. 5). Then the *Saccharum* species diverged from their common ancestors at 1.65, 0.73, 0.52, 0.27, and 0.19 MYA for *S. spontaneum*, *S. barberi*, *S. sinense*, *S. officinarum* and *S. robustum*, respectively.

## Discussion

### Sugarcane GBS analysis resources and pipeline

Compared to traditional marker systems, GBS has drawn a great interest for research because of its high throughput, speed, and decreasing cost. GBS-data analysis pipelines including Tassel-GBS and Stacks were well developed to handle raw reads to final SNP set for diploid species. However, for polyploid species, these GBS pipelines for SNPs calling will need to be optimized considering possible multiple alleles and high dosage alleles present in polyploid species. Sugarcane is an extreme model of polyploid species, and the application of GBS in sugarcane encounters extra difficulty specifically in the downstream sequence analysis. Recently, GBS has been utilized for sugarcane genotyping [20], in which 151 F_1_ individuals derived from a cross between sugarcane cultivars were genotyped for linkage mapping and QTL analysis. The results from Balsalobre et al.’s study demonstrated the powerfulness of GBS for polyploid genetic studies. In our study, we focused on sequence variation mining across different species among the *Saccharum* complex, which were selected from World Collection of Sugarcane and Related species. We described the GBS pipelines that utilized NGS raw reads, regarding reference-based and reference-independent pipelines, alignment methods, and sequencing depth, which were not fully explored in the diverse *Saccharum* complex yet. In total, 76,251 SNPs, 5642 InDels, 6380 PAVs, and 826 CNVs were detected among the 14 *Saccharum* complex accessions. With further validation, these sequence variations could be useful to identify alleles contributing to important agronomic traits specifically derived from interspecific hybridization.

**Fig. 6:**
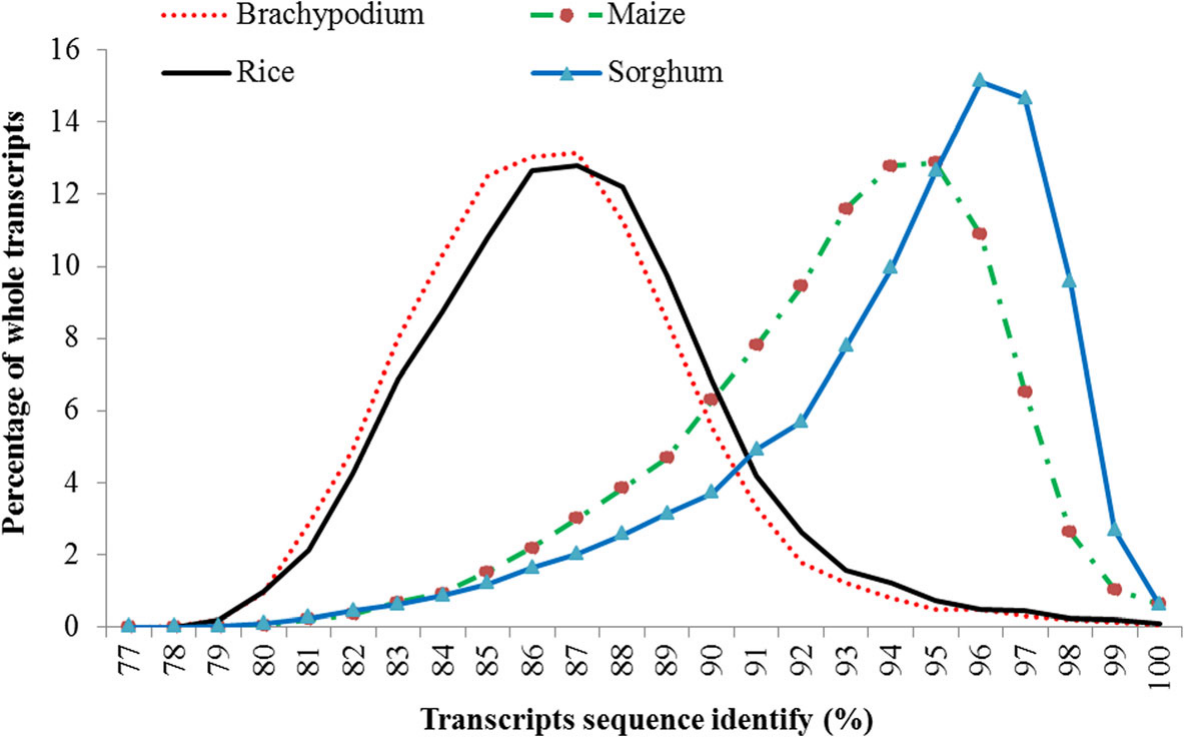
Sequence similarity of sugarcane expressed sequence tags in comparison with transcript sequences of other grass species including *Brachypodium distachyon*, maize, rice, *Setaria viridis* and sorghum

A reference genome sequence for sugarcane has not been available to the research community, even though great international efforts have been invested. Fortunately, the genome of sorghum, one of the closest related diploid crops to sugarcane [23], has been sequenced, well assembled, and is publicly available [21]. The sorghum genome sequence is frequently used as a reference for sugarcane genetic and genomic studies due to the high collinearity between the genomes of sugarcane and sorghum with approximately 95% sequence similarity in the genic regions [7, 17, 24–27]. We further compared a compiled sugarcane ESTs database with whole transcript sets of four grass species, including *Brachypodium distachyon* [28], maize [29], rice [30], and sorghum [21]. Sorghum shared the highest similarity with sugarcane, in which 87.3% sorghum transcripts had corresponding sugarcane ESTs with more than 90% identity (Fig. 6). We also compared the sorghum genome and assembled sugarcane transcripts as reference for our GBS data analysis. The overall unique alignment rate was 32.4% and 20.8% for the sorghum genome and assembled sugarcane transcripts, respectively. Similar results were also showed in the recently published paper [20], in which four references were evaluated. The results showed that the sorghum genome had less aligned sugarcane tags than methyl-filtered sugarcane genome but outperformed RNA-seq sugarcane transcritome and SUCEST project sequences. However, we noticed that there were more than one million scaffords in the methyl-filtered sugarcane genome, which may include some sequences from homo(eo)logous regions not collapsed into a mono-genomic contigs. This speculation was supported by the significantly reduced unique alignment rate against the methyl-filtered sugarcane genome compared with overall alignment rate (58.8% vs. 87.9%). In our study, the percentages of uniquely mapped reads obtained by the GBS ranged from 30.0 to 35.8%, which were quite consistent among the 14 accessions. Though the alignment rates were lower than that in species with reference genome sequences [2, 22, 31], the sorghum genome has been well assembled and annotated, and SNPs called can be easily applied into comparative genomic studies and be associated to the genes if located in the genic regions. Therefore, we suggested using the sorghum genome as the reference for reference-based SNP calling before the sugarcane reference genomes become public available.

Both Bowtie 2 and BWA-backtrack are suitable for aligning short NGS reads and have been widely used for GBS data analysis in other plant species [32–34]. Our results indicated that Bowtie 2 could generate much more unique alignments than BWA-backtrack on short reads (64-bp). This is consistent with previous reports that Bowtie 2 performed better or was more sensitive in detecting alignment than BWA-backtrack on short reads [33]. The BWA-backtrack used an end-to-end alignment model while local alignment was used in Bowtie 2. Local alignment model might optimize the alignment scores by trimming off extreme alignments in the reads. Since we were analyzing sugarcane sequences without its own reference genome, the strategy of local alignment was more applicable than BWA-backtrack. We therefore suggest using Bowtie 2 as the alignment method for GBS data analysis of sugarcane, for which the reference genome is not available. However, a downstream variants calling and application of the variants called based on the two different aligners should be compared to further validate this. In addition, sugarcane reads generated by different genome reduction (targeting) method or with different length may cause different results.

A total of 76,251 non-redundant SNPs were called by the five reference-based callers, which were less than the SNPs called by Stacks de novo, a caller not relying on reference genome sequences. This may be due to the low alignment rate of reads to the genome of sorghum instead of sugarcane. Out of the 78.3 million clean reads, approximately 30% were uniquely mapped to the sorghum genome, approximately 10% were mapped to more than one location, and approximately 60% did not align to the genome. Among 68,706 tags and 10,700 catalogs that were used to call SNPs for the UNEAK and Stacks de novo respectively, only 24.9% of the tags and 25.9% of the catalogs could be uniquely aligned to sorghum genome. This also supported that only a small portion of reads was used to call SNPs in reference-based pipelines. Although the sorghum genome was the best reference for our representative sugarcane germplasm accessions, more than half of the good reads were not used in the reference-based analysis, leading to a large number of sequence variants not identified. Regarding sequence variation detection, the sorghum reference-based methods focused on the conserved regions between sugarcane and sorghum, while the de novo methods extended to sugarcane specific regions. However, we still recommend to use reference-based methods for sugarcane GBS data analysis for three reasons: 1) sequence variants called by reference-based methods could be cross-validated (Fig. 2a), which can increase the accuracy of SNPs called [35]; 2) variants called by reference-based methods can be used for further downstream annotation if the reference genome is fully annotated; and 3) variants called by reference-based methods can distinguish duplicated genes or paralogues, especially in sugarcane with up to 12 sets of chromosomes. Therefore, for non-model species like sugarcane, a set of reliable SNPs should be more important than a set of larger number of SNPs with unknown quality. Nevertheless, if the SNPs called by the non-reference-based methods could be further validated, they can be used to enrich the variants database.

Sequencing depth is an important filter to be set for removing low quality sequence variants, specifically in polyploid species, where high coverage is usually hard to achieve. At a locus with shallow sequence depth, it is easy for SD SNPs of heterozygous genotype to be called as homozygous genotype. For example, an SNP locus with genotype “AAAAAAAAAAAC” can be easily called as homozygous locus with genotype “AAAAAAAAAAAA”, due to randomly sampling of this locus with a low sequence depth. However, SD SNPs are very important as they have a simple inheritance pattern in polyploid species and have been extensively used for linkage analysis. Therefore, we estimated the sequence depth required for the expected number of reads for minor alleles in SD markers to be called, and evaluated the SNPs called at different sequence depths (Fig. 3). Based on the results, we used the sequence depth of 56 reads per sample to select high quality SNPs for downstream analysis. At this depth, we could obtain two reads for minor alleles at SD SNP loci and could have more than 90% of potential SNPs called even for a dodecaploid species. Since we assumed no sequencing error in the clean reads and considered the 4.3 million uniquely mapped reads as a whole read set to call all potential SNPs, we might have underestimated the sequence depth needed. The results of validation of SNPs called and phylogenetic analysis by using the SNPs filtered at this depth were quite consistent with previous researches [36, 37], supporting that this depth should be a good starting point to filter SNPs for diversity species with ploidy levels ranging from 4X to 12X.

### Comparison of GBS and HCS

There are several genome complexity reduction methods for NGS-based sequence variance discovery. To evaluate the efficiency of GBS, we compared the variants called by GBS with one of the target enrichment methods, HCS. The GBS and HCS were widely applied to efficiently discover genome-wide sequence variants after sequencing the reduced genomes in various species [2, 10–17, 38].

The HCS was used to identify sequence variations among 12 of the 14 accessions (Except for CP95–1039 and CP88–1762) [19]. For HCS, 55,946120-mer probes were designed according to the sorghum genome and the sugarcane unigene sets to capture corresponding genomic regions of sugarcane for NGS sequencing. The sequence reads, alignment, coverage and SNPs called by these two methods were compared with comparable settings (Table 3). There were 54 million clean reads (64-bp) generated by the GBS with 96-plex one-lane sequencing and 411.7 million clean reads (89-bp in average) for the HCS with 12-plex one-lane sequencing. The percentages of uniquely mapped reads to sorghum genome of GBS and HCS data were 32.2% and 73.8%, respectively. The low alignment rate of the reads generated by GBS indicated that a large amount (more than half) of the reads from GBS generated from non-coding regions of sugarcane since the coding regions between sugarcane and sorghum genomes were highly similar (95.2% sequence identity) [27]. The GBS and HCS sequence read coverage of the sorghum genome was 4.1 Mb and 115.8 Mb, accounting for 0.6% and 17.6% of the genome, respectively, supporting that both methods worked for non-model species with complex genomes in reducing the genome. The relative small target region of the GBS reads is due by the size selection of 51–500 bp fragments cut by a rare cutter, *Pst*I for sequencing, while the large amount of target regions of HCS reads is related to the number and coverage of probes designed (approximately 56 thousands of 120-mer oligos) to capture the fragments with homology to probe sequences. Thus, the sequence coverage of HCS is highly flexible and controllable depending on the number of probes and target region size of interests, while the sequence coverage of GBS relying on the enzyme used is mostly fixed with minimum adjustment. The average sequence depth per sample was comparable (22.5 vs. 19.0) between the two approaches used in the study, which mainly relied on sequence request instead of methods per se.

**Table 3:** Characters and efficiency comparison between the genotyping by sequencing (GBS) and the hybridization capture sequencing (HCS) for next generation sequencing enabled high throughput sugarcane genotyping (M: million)

	GBS	HCS
Clean reads (M)/uniquely mapped reads (M) (%)	54.0/17.4 (32.2%)	411.7/304.0 (73.8%)
Uniquely mapped reads in genic regions (M) (%)	14.5 (83.3%)	277.4 (67.4%)
Uniquely mapped reads in inter-genic regions (M) (%)	2.9 (16.7%)	143.4 (32.6%)
Coverage for sorghum genome (Mb) (%)	4.1 (0.6%)	115.8 (17.6%)
Coverage for genic regions (Mb) (%)	3.0 (73.2%)	70.5 (58.4%)
Coverage for inter-genic regions (M) (%)	1.1(26.8%)	45.3 (39.1%)
Total SNPs/density of coverage (per Mb)	20,691/5046.6	884,287/7636.2
SNPs in genic regions (%) /intergenic regions (%)	17,550 (84.8%)/3141 (15.2%)	799,950 (90.5%)/84,337 (9.5%)
Cost per sample/cost per thousand SNPs	$388/$0.4	$70/$3.4

**Fig. 7:**
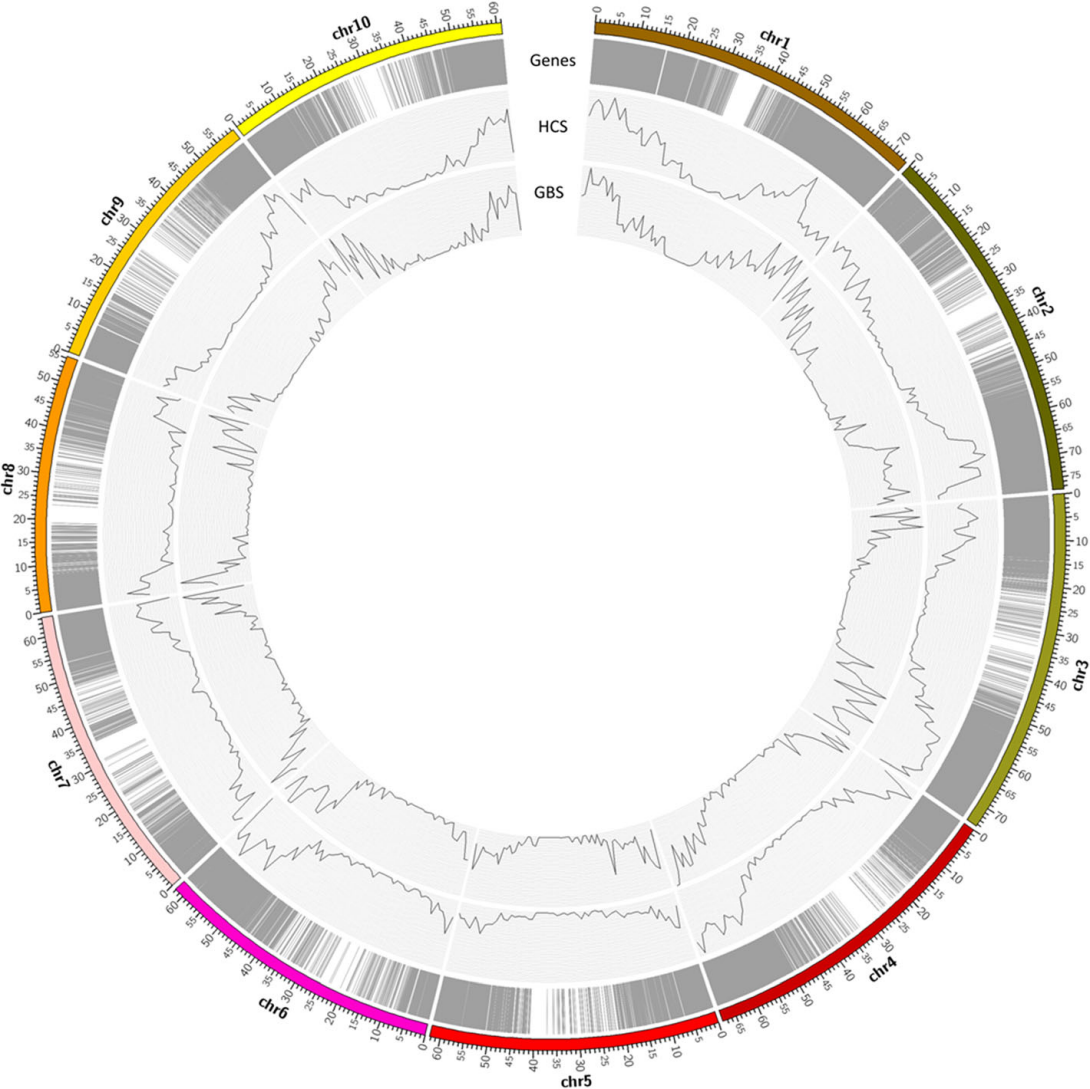
Genome-wide distribution of genes, single nucleotide polymorphisms (SNPs) called by the hybridization capture sequencing (HCS) and the genoytyping by sequencing (GBS) according to sorghum genome 3.0

SNPs called and filtered using the same pipeline (Samtools) from GBS and HCS were 20,691 and 884,287 SNPs respectively, and 1478 were concordantly SNPs between the two methods. The SNP densities from the GBS and HCS were 5047 and 7636 SNPs per Mb over the sequence covered sorghum genome. The slight difference of SNP density in GBS and HCS may be caused by their different sequence coverage. The genome-wide distribution of SNPs showed a similar pattern that was highly enriched at gene-rich regions according to the sorghum genome (Fig. 7), with 84.8 and 90.5% in genic regions for the GBS approach and the HCS approach, respectively (Table 3). Most of the SNPs were in genic regions from the HCS method because the majority of probes were designed from genic region to the target gene sequences [19]. For GBS, the portion of SNPs located in genic region is also very high (84.8%). This may occur because the reads generated from inter-genic regions may not align well to the sorghum genome. More than 60% of the good GBS reads in this study did not align to the sorghum genome, a high portion of which may come from the inter-genic regions. Thus, to fully utilize the data generated by GBS, secondary non-reference-based SNP calling procedures are necessary or a better reference genome is needed. In our study, the non-reference-based methods, UNEAK and Stacks de novo, were used for SNP calling to fully identify the potential SNPs exist.

In terms of cost of per sample, HCS is higher than GBS ($388 vs. $70). However, in terms of cost of per thousand of SNPs generated from the same reference-based caller, the price is lower for HCS with $0.4 compared to $3.4 for GBS. It should be noticed that additional large number of SNPs can be called from GBS data using non-reference based callers, which can significantly complement the number of SNPs generated by GBS method. Generally, GBS could be good to discover SNPs for linkage analysis and QTL mapping, which do not rely on reference genome sequences and do not require high density of markers because of normally high linkage disequilibrium in the bi-parental cross populations for mapping. The HCS requires prior availability of a reference genome or enriched EST database of the species for specific probe design. Reads generated by HCS are mostly from genic region. HCS with a large number of probes is suitable for high-density marker development. The small overlapping (2.6%) of the sequence covered regions between GBS and HCS indicated that the two methods could compensate each other in order to increase the marker coverage on the genome.

### Heterozygosity, single dose SNPs, and transition/transversion ratios in polyploid species

In polyploid species, multiple alleles and different dosages exist. In the 14 accessions, SD SNPs ranged from 38.3 to 62.3% and were the most abundant SNPs compared to duplex, triplex, and other multiple dosage SNPs. The percentage of SD SNPs decreased with the increasing ploidy levels, which is consistent with Garcia’s report [39], and could be due to the high heterozygosity in highly polyploid hybrids. SD markers are the most important and efficiently used markers in polyploid species [40]. Utilizing SD SNPs in polyploid species is prevalent and critical in the linkage analysis and QTL mapping because of their simple inheritance and easy detection. The large number of SD SNPs identified in this study will be highly applicable for sugarcane linkage and QTL analyses.

For reliable SNP calling, specifically for SD SNP identification, we suggest to filter the SNP at a depth of 56, a depth that could obtain two reads for the SD minor alleles and could call 90% of potential SNPs (Fig. 3). However, with the suggested depth for SNP filtering, the chance to call a genotype with SD SNPs as a ‘homozygous genotype’ would be higher in dodecaploids than in hexaploids, since the required read depth to ensure reliable SD SNP calling depends on the ploidy level. In order to evaluate whether there was such bias in our observation, we analyzed the percentage of SD SNPs in dodecaploids by filtering SNPs using a higher depth of 110 as a cutoff per sample. The percentage of single dose SNPs was not reduced significantly (42.1% vs. 41.6%), suggesting that the reduced proportion of SD SNPs in dodecaploids may be due to the high ploidy level instead of the sequence depth. We also observed that the percentages of multiple allelic SNPs and high dosage SNPs in dodecaploids were higher than in hexaploids and octoploids (Fig. 4a), which supported our hypothesis. Assuming equal mutation rate, it is easy to deduce that the chance for SD SNPs changed to other dosages is higher in individuals with high polyploid level than with low ploidy level.

When mutation occurs randomly, the theoretical Ts/Tv ratio should be 1:2 with no bias towards transition or transversion substitution. A Ts/Tv ratio greater than 1:2 indicates that transition mutation is favored over transversion during evolution, which was observed in many plant species. For example, the Ts/Tv ratio was 2.5 in maize [41], 1.45 in *Setaria viridis* [42], 1.82 in *Populus tremula* [43], 2.4 in *Arabidopsis thaliana* [44], 1.65 in eggplant [45], 1.8 in flax [46], and 1.52 in *Brassica napus* L [47], with a big variation between different species. In our study, the estimated average Ts/Tv ratios was 1.63 in the 14 accessions based on genome-wide SNPs with a range of 1.55 in the *Erianthus* genus to 1.66 in the *Saccharum* genus. The different Ts/Tv ratios of these two genera reflect their different evolutionary force and status, which might have been influenced by different environmental conditions.

### Divergence of difference species in the *Saccharum* Complex

Based on the phytogeographical data, morphological similarity, cytology, and breeding behavior, Mukherjee (1957) proposed that the *Saccharum* complex should include four genera: *Saccharum, Erianthus, Sclerostachya*, and *Narenga* [48]. Daniels et al. (1975) added *Miscanthus* into the *Saccharum* complex later on [48–50]. With the availability of molecular data, origins and genetic relationships of these genera in the *Saccharum* complex can be further characterized. Our results demonstrated that the *Erianthus* species were distantly related to the *Saccharum* species, which is consistent with previous reports [37]. The genera *Erianthus* and *Saccharum* diverged more than 10 MYA, while the divergence time among the *Saccharum* species ranged from 0.19 to 1.65 MYA. The genetic distance between the *Saccharum* genus and the *Erianthus* genus is even larger than that between the *Saccharum* genus and sorghum. *S. barberi*, *S. sinense*, and *Saccharum* hybrids were placed between *S. spontaneum* and *S. officinarum*, supporting their origin from interspecific hybridization between *S. spontaneum* and *S. officinarum* [37, 51, 52]. *S. robustum* clustered with *S. officinarum*, supporting *S. robustum* as the immediate ancestor of *S. officinarum* [36, 37].

## Conclusion

The GBS pipelines including the reference sequences, alignment methods, sequence variant callers, and sequence depth were recommended and discussed for the *Saccharum* complex and other related species. A large number of sequence variations were discovered in the *Saccharum* complex, including SNPs, InDels, PAVs, and CNVs, which can be further validated and utilized to identify alleles contributing to important agronomic traits. Genome-wide SNPs were further used to study sequence features of polyploid species and divergence of the *Saccharum* complex. The results demonstrated that GBS can be an effective approach to discover genomic sequence variations in highly polyploid and heterozygous species.

## Methods

### Plant materials

Fourteen representative germplasm accessions in *Saccharum* complex (Table 1) were selected to study sequence variations in the high ploidy sugarcane. Twelve of the 14 accessions were selected from the World Collection of Sugarcane and Related Grass after genotypic and phenotypic evaluation [53], and the other two were *S.* hybrids, used as parental lines of a segregating mapping population. Specifically, the 14 selected accessions included two accessions of *S. officinarum* (P-MAG-84, NG96–024), two accessions of *S. spontaneum* (SES196, IND81–14), one accession of *S. barberi* (Pathri), one accession of *S. robustum* (NG57–054), one accession of *S. sinense* (TekchaOk), one accession of *Erianthus procerum* (Kalimpon), one accession of *E. rufipilu* (US57–060), one accession in the core collection with species name undetermined (US61–037), and four accessions of *Saccharum* hybrids (Q050, R570, CP95–1039, CP88–1762), in which CP95–1039 and CP88–1762 are the parental lines of a mapping population for another study.

### DNA isolation and sequencing

Total genomic DNA was isolated from leaf samples using the CTAB method with minor modifications [27]. DNA concentration was measured using a Quant-iT PicoGreen dsDNA Assay Kit. Only DNA with a good quality and a concentration higher than 50 ng/μl was used for GBS library preparation. The reduced representation libraries and sequencing were performed at the Institute of Genomic Diversity, Cornell University, following the optimized GBS protocol [2]. In brief, DNA samples were digested with *Pst*I, a restriction enzyme with a rare cutting site (CTGCAG). Libraries of 96-plex (95 DNA samples and one negative control) were constructed using the well-optimized protocol, and then sequenced on the Illumina HiSeq 2000 platform. To increase the sequence depth, the 96-plex libraries were sequenced twice in total.

### Sequence trimming and mapping

Raw sequence reads were filtered to select reads with barcodes and expected remnant of the *Pst*I cutting site (CTGCAG). The clean reads (SRP082222) were then trimmed to 64-bp for further sequence analysis. Clean reads shorter than 64-bp were padded with “A” at the end to make the length up to 64-bp. For evaluation of alignment efficiency, we compared two different aligners: BWA version 0.7.12 (http://sourceforge.net/projects/bio-bwa/files/) and Bowtie 2 version 2.2.5 (http://sourceforge.net/projects/bowtie-bio/files/bowtie2/2.2.5/) to map clean reads from a hybrid, CP88–1762 to sorghum genome version 3.0 [21, 33, 34]. Bowtie 2 was used with default --local mode. BWA-backtrack was run with default settings. Uniquely mapped reads were extracted from SAM files following the methods described by Clevenger et al. [54].

### Sequence variant calling

Different variant callers were used to identify SNPs and InDels including reference-based callers, Tassel [32], Stacks [55], Samtools [56], Genome Analysis ToolKit (GATK) [57], and Freebayes [58], and non-reference-based callers, the universal network enabled analysis kit (UNEAK) pipeline in Tassel and Stacks de novo [55, 59]. For reference-based callers, only uniquely mapped reads were used for sequence variation detection. In Tassel pipeline, identical clean reads formed a GBS tag and the tags with a minimum count of five were used for genotyping. Stacks pipeline was run with the parameters -m 3 -n 3. In Stacks, identical clean reads with a minimum depth of three reads formed a stack and stacks from the same locus were merged into a catalog. Samtools pipeline was used to call variants with the parameters -ugf. Freebayes pipeline was run with the parameters -F 0.05–0 -q 20 --min-coverage 5 -p (ploidy level). GATK pipeline was used with settings: -T UnifiedGenotyper -glm BOTH -ploidy (ploidy level) -mbq 20 -stand_call_conf 30 -stand_emit_conf 10. Samtools, GATK and Freebayes were used to call SNPs and InDels. Sequence variants called were filtered with a minimum depth of 35 per sample. To discover single dose (SD) SNPs, genotypes of SNPs were first called for each accession using GATK and Freebayes by setting ploidy levels according to the estimation (Table 1) [19]. The SD SNPs of each accession were then identified from bi-allelic SNPs with one of the alleles in one copy according to their genotypes called. For the bi-allelic SNPs, the allele with less dosage in the genotype than the other allele was defined as the minor allele, while the other alternative allele with more dosage in the genotype was defined as the major allele.

Only sequence regions with *Pst*I digestion site and a digested fragment size between 51 to 500 bp were selected for detection of presence-absence variations (PAVs) and copy number variations (CNVs). The PAVs were identified following the criteria 1) no reads aligned to at least one accession; 2) a minimum of five reads aligned to at least one accession. To eliminate the influence of different sequence depth of each sample, the depth of coverage was normalized using the formula: read depth of targeting region = [10^9^ * (no. of reads in the region)] / [(Total mapped reads) * (length of the region)]. Then log_2_ ratio of normalized reads of each two accessions were calculated and used to assess the CNVs. The targeting regions were considered as CNVs with log_2_ ratios falling outside of the 99% range (mean ± 3sd) of log2 ratio.

### Sequence depth evaluation for genotype calling

The number of reads for alleles at a SNP locus follows a binomial distribution [60]. Thus, for a given bi-allelic SNP (A/B) with alleles ‘A’ and ‘B’, the probability to obtain k reads for allele ‘A’ can be calculated using the following formula:

$$ P\left(\mathrm{A}|\mathrm{AB}\right)=1-{\sum}_{i=0}^{k-1}\left(\genfrac{}{}{0pt}{}{n}{i}\right){x}^i{\left(1-x\right)}^{n-i} $$

Where n = the total of sequencing reads, x = the frequency of allele ‘A’, k = the number of reads for allele (A), and *P* = the probability to obtain k reads for allele ‘A’. Based on this formula, the sequence depth was estimated in order to obtain the expected number of reads for minor alleles of SD SNPs with a probability higher than 95% at different ploidy levels. For simplicity of the estimation, we assumed no sequencing error in the clean reads.

Uniquely mapped reads of accession, CP88–1762, were used to determine the relationship of sequence depth and percentage of SNPs called. The data set with the uniquely mapped reads was considered as a whole sequence set that could call all potential SNPs. Eight sub-data sets with 20, 30, 40, 50, 60, 70, 80, and 90% of the whole sequence set were randomly sub-sampled for 10 times, separately. Then SNPs were called in these 10 times of eight sub-sampled data sets. Compared with the whole sequence set, the percentages of SNPs called were calculated for each of the eight sub-sampled data sets.

### Validation of single nucleotide polymorphisms

A total of 16 SNPs called with a minimum sequence depth of 56 per locus per sample (Table 2) were randomly selected for validation. Primers were designed using BatchPrimer3 v1.0 (https://probes.pw.usda.gov/batchprimer/) from sorghum genome harboring the SNPs. Only the primers with one alignment to the sorghum genome 3.0 [21] and alignment to sugarcane contigs [19] with less than two mismatches were picked. Out of the 14 accessions, eight were used for validation, including SES196, NG57–054, TekchaOk, Pathri, NG96–024, R570, CP88–1762, and CP95–1039. PCR products generated from the eight samples were directly sequenced by Sanger sequencing platform at the Interdisciplinary Center for Biotechnology Research (ICBR) at the University of Florida.

### Sequence variation features

Concordantly called SNPs by using GATK and Freebayes with a minimum depth of 56 were used to calculate the percentages of heterozygosity, SD SNPs, multiple allelic SNPs, and transition/transversion (Ts/Tv) ratios for each of the 14 accessions respectively. The Ts/Tv ratio was determined by SnpEff (v4.1) [61]. The percentages of heterozygosity, the percentage of SD SNPs and the percentage of multiple allelic SNPs were calculated following the formulas below,

$$ \mathrm{Heterozygosity}\ \left(\%\right)={\left(\mathrm{number}\  \mathrm{of}\  \mathrm{heterozygous}\  \mathrm{loci}/\mathrm{total}\  \mathrm{number}\  \mathrm{of}\  \mathrm{loci}\right)}^{\ast }100 $$

$$ \mathrm{SD}\ \mathrm{SNPs}\ \left(\%\right)={\left(\mathrm{number}\  \mathrm{of}\ \mathrm{SD}\ \mathrm{SNPs}/\mathrm{the}\  \mathrm{total}\  \mathrm{number}\  \mathrm{of}\  \mathrm{SNPs}\right)}^{\ast }100 $$

$$ \mathrm{Multiple}\  \mathrm{allelic}\  \mathrm{SNPs}\ \left(\%\right)={\left(\mathrm{number}\  \mathrm{of}\  \mathrm{multiple}\  \mathrm{allelic}\  \mathrm{SNPs}/\mathrm{the}\  \mathrm{total}\  \mathrm{number}\  \mathrm{of}\  \mathrm{SNPs}\right)}^{\ast }100 $$

### Divergence analysis

Concordantly called SNPs by five reference-based callers with a minimum depth of 56 were used for phylogenetic analysis of the 14 accessions. A divergence time tree for the 14 accessions and sorghum was constructed using the molecular evolutionary genetic analysis software version 6.0 (MEGA 6.0) [62, 63]. First, the best substitution model was chosen using the feature ‘Find Best DNA/Protein Models (ML)’ of MEGA 6. Then maximum likelihood method and the best model “T92” were applied to construct a phylogenetic tree with 1000 bootstrap replicates. Finally, the obtained tree was used as a starting tree for the time tree construction using ‘RealTime-ML’ feature in MEGA 6. The divergence time of sorghum and R570 was used as a calibration for this analysis [27].

## Abbreviations

CNV: Copy number variation
GBS: Genotyping by sequencing
GWAS: Genome wide association study
HCS: Hybridization capture sequencing
MYA: Million years ago
NGS: Next generation sequencing
PAV: Presence/absence variant
PCR: Polymerase chain reaction
QTL: Quantitative trait locus
SD: Single dose
SNP: Single nucleotide polymorphism
Ts/Tv: Transition/transversion

## References

[1] Mardis ER (2008). The impact of next-generation sequencing technology on genetics. Trends Genet.

[2] Elshire RJ, Glaubitz JC, Sun Q, Poland JA, Kawamoto K, Buckler ES, Mitchell SE (2011). A robust, simple genotyping-by-sequencing (GBS) approach for high diversity species. PLoS One.

[3] Li R, Li Y, Fang X, Yang H, Wang J, Kristiansen K, Wang J (2009). SNP detection for massively parallel whole-genome resequencing. Genome Res.

[4] Smith DR, Quinlan AR, Peckham HE, Makowsky K, Tao W, Woolf B, Shen L, Donahue WF, Tusneem N, Stromberg MP, Stewart DA, Zhang L, Ranade SS, Warner JB, Lee CC, Coleman BE, Zhang Z, McLaughlin SF, Malek JA, Sorenson JM, Blanchard AP, Chapman J, Hillman D, Chen F, Rokhsar DS, McKernan KJ, Jeffries TW, Marth GT, Richardson PM (2008). Rapid whole-genome mutational profiling using next-generation sequencing technologies. Genome Res.

[5] Varshney RK, Nayak SN, May GD, Jackson SA (2009). Next-generation sequencing technologies and their implications for crop genetics and breeding. Trends Biotechnol.

[6] D'Hont A (2005). Unraveling the genome structure of polyploids using FISH and GISH; examples of sugarcane and banana. Cytogenet Genome Res.

[7] Davey JW, Hohenlohe PA, Etter PD, Boone JQ, Catchen JM, Blaxter ML (2011). Genome-wide genetic marker discovery and genotyping using next-generation sequencing. Nat Rev Genet.

[8] Mamanova L, Coffey AJ, Scott CE, Kozarewa I, Turner EH, Kumar A, Howard E, Shendure J, Turner DJ (2010). Target-enrichment strategies for next-generation sequencing. Nat Methods.

[9] Turner EH, Ng SB, Nickerson DA, Shendure J (2009). Methods for genomic partitioning. Annu Rev Genomics Hum Genet.

[10] Everett MV, Seeb JE (2014). Detection and mapping of QTL for temperature tolerance and body size in Chinook salmon (*Oncorhynchus tshawytscha*) using genotyping by sequencing. Evol Appl.

[11] He J, Zhao X, Laroche A, Lu Z, Liu H, Li Z (2014). Genotyping-by-sequencing (GBS), an ultimate marker-assisted selection (MAS) tool to accelerate plant breeding. Front Plant Sci.

[12] Narum SR, Buerkle CA, Davey JW, Miller MR, Hohenlohe PA (2013). Genotyping-by-sequencing in ecological and conservation genomics. Mol Ecol.

[13] Poland JA, Brown PJ, Sorrells ME, Jannink JL (2012). Development of high-density genetic maps for barley and wheat using a novel two-enzyme genotyping-by-sequencing approach. PLoS One.

[14] Poland JA, Rife TW (2012). Genotyping-by-sequencing for plant breeding and genetics. The Plant Genome.

[15] Uitdewilligen JG, Wolters AA, Bjorn B, Borm TJ, Visser RG, van Eck HJ (2013). A next-generation sequencing method for genotyping-by-sequencing of highly heterozygous autotetraploid potato. PLoS ONE.

[16] Ward JA, Bhangoo J, Fernandez-Fernandez F, Moore P, Swanson JD, Viola R, Velasco R, Bassil N, Weber CA, Sargent DJ (2013). Saturated linkage map construction in *Rubus idaeus* using genotyping by sequencing and genome-independent imputation. BMC Genomics.

[17] Bundock PC, Casu RE, Henry RJ (2012). Enrichment of genomic DNA for polymorphism detection in a non-model highly polyploid crop plant. Plant Biotech J.

[18] Grativol C, Regulski M, Bertalan M, McCombie WR, Silva FR, Zerlotini Neto A, Vicentini R, Farinelli L, Hemerly AS, Martienssen RA (2014). Sugarcane genome sequencing by methylation filtration provides tools for genomic research in the genus *Saccharum*. Plant J.

[19] Song J, Yang X, Resende MF, Neves LG, Todd J, Zhang J, Comstock J, Wang J (2016). Natural allelic variations in highly polyploid *Saccharum* Complex. Front Plant Sci.

[20] Balsalobre TWA, da Silva PG, Margarido GRA, Gazaffi R, Barreto FZ, Anoni CO, Cardoso-Silva CB, Costa EA, Mancini MC, Hoffmann HP (2017). GBS-based single dosage markers for linkage and QTL mapping allow gene mining for yield-related traits in sugarcane. BMC Genomics.

[21] Paterson AH, Bowers JE, Bruggmann R, Dubchak I, Grimwood J, Gundlach H, Haberer G, Hellsten U, Mitros T, Poliakov A, Schmutz J, Spannagl M, Tang H, Wang X, Wicker T, Bharti AK, Chapman J, Feltus FA, Gowik U, Grigoriev IV, Lyons E, Maher CA, Martis M, Narechania A, Otillar RP, Penning BW, Salamov AA, Wang Y, Zhang L, Carpita NC, Freeling M, Gingle AR, Hash CT, Keller B, Klein P, Kresovich S, MC MC, Ming R, Peterson DG, Mehboob-ur-Rahman WD, Westhoff P, Mayer KF, Messing J, Rokhsar DS (2009). The *Sorghum bicolor* genome and the diversification of grasses. Nature.

[22] Heffelfinger C, Fragoso CA, Moreno MA, Overton JD, Mottinger JP, Zhao H, Tohme J, Dellaporta SL (2014). Flexible and scalable genotyping-by-sequencing strategies for population studies. BMC Genomics.

[23] Guimaraes CT, Sills GR, Sobral BW (1997). Comparative mapping of Andropogoneae: *Saccharum* L. (sugarcane) and its relation to sorghum and maize. Proc Natl Acad Sci U S A.

[24] de Setta N, Monteiro-Vitorello CB, Metcalfe CJ, Cruz GM, Del Bem LE, Vicentini R, Nogueira FT, Campos RA, Nunes SL, Turrini PC, Vieira AP, Ochoa Cruz EA, Correa TC, Hotta CT, de Mello Varani A, Vautrin S, da Trindade AS, de Mendonca Vilela M, Lembke CG, Sato PM, de Andrade RF, Jr Nishiyama MY, Cardoso-Silva CB, Scortecci KC, Garcia AA, Carneiro MS, Kim C, Paterson AH, Berges H, D’Hont A, de Souza AP, Souza GM, Vincentz M, Kitajima JP, Van Sluys MA (2014). Building the sugarcane genome for biotechnology and identifying evolutionary trends. BMC Genomics.

[25] Draye X, Lin YR, Qian XY, Bowers JE, Burow GB, Morrell PL, Peterson DG, Presting GG, Ren SX, Wing RA, Paterson AH (2001). Toward integration of comparative genetic, physical, diversity, and cytomolecular maps for grasses and grains, using the sorghum genome as a foundation. Plant Physiol.

[26] Figueira TR, Okura V, Rodrigues da Silva F, Jose da Silva M, Kudrna D, Ammiraju JS, Talag J, Wing R, Arruda P (2012). A BAC library of the SP80–3280 sugarcane variety (*Saccharum* sp.) and its inferred microsynteny with the sorghum genome. BMC Res Notes.

[27] Wang J, Roe B, Macmil S, Yu Q, Murray JE, Tang H, Chen C, Najar F, Wiley G, Bowers J, Van Sluys MA, Rokhsar DS, Hudson ME, Moose SP, Paterson AH, Ming R (2010). Microcollinearity between autopolyploid sugarcane and diploid sorghum genomes. BMC Genomics.

[28] International Brachypodium Initiative (2010). Genome sequencing and analysis of the model grass *Brachypodium distachyon*. Nature.

[29] Schnable PS, Ware D, Fulton RS, Stein JC, Wei F, Pasternak S, Liang C, Zhang J, Fulton L, Graves TA, Minx P, Reily AD, Courtney L, Kruchowski SS, Tomlinson C, Strong C, Delehaunty K, Fronick C, Courtney B, Rock SM, Belter E, Du F, Kim K, Abbott RM, Cotton M, Levy A, Marchetto P, Ochoa K, Jackson SM, Gillam B, Chen W, Yan L, Higginbotham J, Cardenas M, Waligorski J, Applebaum E, Phelps L, Falcone J, Kanchi K, Thane T, Scimone A, Thane N, Henke J, Wang T, Ruppert J, Shah N, Rotter K, Hodges J, Ingenthron E, Cordes M, Kohlberg S, Sgro J, Delgado B, Mead K, Chinwalla A, Leonard S, Crouse K, Collura K, Kudrna D, Currie J, He R, Angelova A, Rajasekar S, Mueller T, Lomeli R, Scara G, Ko A, Delaney K, Wissotski M, Lopez G, Campos D, Braidotti M, Ashley E, Golser W, Kim H, Lee S, Lin J, Dujmic Z, Kim W, Talag J, Zuccolo A, Fan C, Sebastian A, Kramer M, Spiegel L, Nascimento L, Zutavern T, Miller B, Ambroise C, Muller S, Spooner W, Narechania A, Ren L, Wei S, Kumari S, Faga B, Levy MJ, McMahan L, Van Buren P, Vaughn MW, Ying K, Yeh CT, Emrich SJ, Jia Y, Kalyanaraman A, Hsia AP, Barbazuk WB, Baucom RS, Brutnell TP, Carpita NC, Chaparro C, Chia JM, Deragon JM, Estill JC, Fu Y, Jeddeloh JA, Han Y, Lee H, Li P, Lisch DR, Liu S, Liu Z, Nagel DH, McCann MC, SanMiguel P, Myers AM, Nettleton D, Nguyen J, Penning BW, Ponnala L, Schneider KL, Schwartz DC, Sharma A, Soderlund C, Springer NM, Sun Q, Wang H, Waterman M, Westerman R, Wolfgruber TK, Yang L, Yu Y, Zhang L, Zhou S, Zhu Q, Bennetzen JL, Dawe RK, Jiang J, Jiang N, Presting GG, Wessler SR, Aluru S, Martienssen RA, Clifton SW, WR MC, Wing RA, Wilson RK (2009). The B73 maize genome: complexity, diversity, and dynamics. Science.

[30] Ouyang S, Zhu W, Hamilton J, Lin H, Campbell M, Childs K, Thibaud-Nissen F, Malek RL, Lee Y, Zheng L, Orvis J, Haas B, Wortman J, Buell CR (2007). The TIGR Rice genome annotation resource: improvements and new features. Nucleic Acids Res.

[31] Jordan KW, Wang S, Lun Y, Gardiner LJ, MacLachlan R, Hucl P, Wiebe K, Wong D, Forrest KL, Consortium IWGS, Sharpe AG, Sidebottom CH, Hall N, Toomajian C, Close T, Dubcovsky J, Akhunova A, Talbert L, Bansal UK, Bariana HS, Hayden MJ, Pozniak C, Jeddeloh JA, Hall A, Akhunov E (2015). A haplotype map of allohexaploid wheat reveals distinct patterns of selection on homoeologous genomes. Genome Biol.

[32] Glaubitz JC, Casstevens TM, Lu F, Harriman J, Elshire RJ, Sun Q, Buckler ES (2014). TASSEL-GBS: a high capacity genotyping by sequencing analysis pipeline. PLoS One.

[33] Langmead B, Salzberg SL (2012). Fast gapped-read alignment with bowtie 2. Nat Methods.

[34] Li H, Durbin R (2010). Fast and accurate long-read alignment with burrows-wheeler transform. Bioinformatics.

[35] Yu X, Sun S (2013). Comparing a few SNP calling algorithms using low-coverage sequencing data. BMC Bioinformatics.

[36] Burnquist W, Sorrelles M, Tanksley S (1995). Characterization of genetic variability in *Saccharum* germplasm by means of restriction fragment length polymorphism (RFLP) analysis. Proc Int Soc Sugar Cane Technol.

[37] Suman A, Ali K, Arro J, Parco AS, Kimbeng CA, Baisakh N (2012). Molecular diversity among members of the *Saccharum* Complex assessed using TRAP markers based on lignin-related genes. BioEnergy Research.

[38] Neves LG, Davis JM, Barbazuk WB, Kirst M (2013). Whole-exome targeted sequencing of the uncharacterized pine genome. Plant J.

[39] Garcia AA, Mollinari M, Marconi TG, Serang OR, Silva RR, Vieira ML, Vicentini R, Costa EA, Mancini MC, Garcia MO, Pastina MM, Gazaffi R, Martins ER, Dahmer N, Sforca DA, Silva CB, Bundock P, Henry RJ, Souza GM, van Sluys MA, Landell MG, Carneiro MS, Vincentz MA, Pinto LR, Vencovsky R, Souza AP (2013). SNP genotyping allows an in-depth characterisation of the genome of sugarcane and other complex autopolyploids. Sci Rep.

[40] Wu K, Burnquist W, Sorrells M, Tew T, Moore P, Tanksley S (1992). The detection and estimation of linkage in polyploids using single-dose restriction fragments. Theor Appl Genet.

[41] Jiao Y, Zhao H, Ren L, Song W, Zeng B, Guo J, Wang B, Liu Z, Chen J, Li W, Zhang M, Xie S, Lai J (2012). Genome-wide genetic changes during modern breeding of maize. Nat Genet.

[42] Huang P, Feldman M, Schroder S, Bahri BA, Diao X, Zhi H, Estep M, Baxter I, Devos KM, Kellogg EA (2014). Population genetics of *Setaria viridis*, a new model system. Mol Ecol.

[43] Ingvarsson PK (2008). Multilocus patterns of nucleotide polymorphism and the demographic history of *Populus tremula*. Genetics.

[44] Ossowski S, Schneeberger K, Lucas-Lledo JI, Warthmann N, Clark RM, Shaw RG, Weigel D, Lynch M (2010). The rate and molecular spectrum of spontaneous mutations in *Arabidopsis thaliana*. Science.

[45] Barchi L, Lanteri S, Portis E, Acquadro A, Vale G, Toppino L, Rotino GL (2011). Identification of SNP and SSR markers in eggplant using RAD tag sequencing. BMC Genomics.

[46] Kumar S, You FM, Cloutier S (2012). Genome wide SNP discovery in flax through next generation sequencing of reduced representation libraries. BMC Genomics.

[47] Clarke WE, Parkin IA, Gajardo HA, Gerhardt DJ, Higgins E, Sidebottom C, Sharpe AG, Snowdon RJ, Federico ML, Iniguez-Luy FL (2013). Genomic DNA enrichment using sequence capture microarrays: a novel approach to discover sequence nucleotide polymorphisms (SNP) in *Brassica napus* L. PLoS One.

[48] Mukherjee SK. Origin and distribution of *Saccharum*. Bot Gaz. 1957:55–61.

[49] Amalraj VA, Balasundaram N (2006). On the taxonomy of the members of ‘*Saccharum* Complex’. Genet Resour Crop Evol.

[50] Daniels J, Smith P, Paton N, Williams CA (1975). The origin of the genus *Saccharum*. Sugarcane Breed News.

[51] D'Hont A, Paulet F, Glaszmann JC (2002). Oligoclonal interspecific origin of 'North Indian' and 'Chinese' sugarcanes. Chromosom Res.

[52] Piperidis G, Piperidis N, D'Hont A (2010). Molecular cytogenetic investigation of chromosome composition and transmission in sugarcane. Mol Gen Genomics.

[53] Nayak SN, Song J, Villa A, Pathak B, Ayala-Silva T, Yang X, Todd J, Glynn NC, Kuhn DN, Glaz B (2014). Promoting utilization of *Saccharum* spp. Genetic Resources through Genetic Diversity Analysis and Core Collection Construction. PLoS One.

[54] Clevenger J, Chavarro C, Pearl SA, Ozias-Akins P, Jackson SA (2015). Single nucleotide polymorphism identification in Polyploids: a review, example, and recommendations. Mol Plant.

[55] Catchen J, Hohenlohe PA, Bassham S, Amores A, Cresko WA (2013). Stacks: an analysis tool set for population genomics. Mol Ecol.

[56] Li H, Handsaker B, Wysoker A, Fennell T, Ruan J, Homer N, Marth G, Abecasis G, Durbin R (2009). 1000 genome project data processing subgroup: the sequence alignment/map format and SAMtools. Bioinformatics.

[57] McKenna A, Hanna M, Banks E, Sivachenko A, Cibulskis K, Kernytsky A, Garimella K, Altshuler D, Gabriel S, Daly M, DePristo MA (2010). The genome analysis Toolkit: a MapReduce framework for analyzing next-generation DNA sequencing data. Genome Res.

[58] Garrison E, Marth G: Haplotype-based variant detection from short-read sequencing. arXiv preprint arXiv 2012, 1207: 3907.

[59] Lu F, Lipka AE, Glaubitz J, Elshire R, Cherney JH, Casler MD, Buckler ES, Costich DE (2013). Switchgrass genomic diversity, ploidy, and evolution: novel insights from a network-based SNP discovery protocol. PLoS Genet.

[60] Li X, Wei Y, Acharya A, Jiang Q, Kang J, Brummer EC (2014). A saturated genetic linkage map of autotetraploid alfalfa (*Medicago sativa* L.) developed using genotyping-by-sequencing is highly syntenous with the *Medicago truncatula* genome. G3 (Bethesda).

[61] Cingolani P, Platts A, Wang le L, Coon M, Nguyen T, Wang L, Land SJ, Lu X, Ruden DM (2012). A program for annotating and predicting the effects of single nucleotide polymorphisms, SnpEff: SNPs in the genome of *Drosophila melanogaster* strain w1118; iso-2; iso-3. Fly (Austin).

[62] Tamura K, Battistuzzi FU, Billing-Ross P, Murillo O, Filipski A, Kumar S (2012). Estimating divergence times in large molecular phylogenies. Proc Natl Acad Sci U S A.

[63] Tamura K, Stecher G, Peterson D, Filipski A, Kumar S (2013). MEGA6: molecular evolutionary genetics analysis version 6.0. Mol Biol Evol.

